# Vacancy in Transylvania University

**Published:** 1845-10

**Authors:** 


### VACANCY IN TRANSYLVANIA UNIVERSITY.

From the following notice it will be perceived, that the appointment of Professor Richardson's successor is not to take place before the last of January next, and that the instruction in that chair is to be given, in the interim, by Dr. Mitchell, the Professor of Materia Medica.

"*To the Medical Public.*—The chair of Obstetrics and the Diseases of Women and Children in the Medical Department of Transylvania University, is at present vacant; and with a view to fill it in the best possible manner, applications for the place are invited from the members of the Medical Profession. Communications on the subject must be forwarded to the Dean of the Medical Faculty prior to the 30th day of January next, when the appointment will be made. It will be required, in conformity with a resolution of the Board of Trustees, that the person selected shall make Lexington his permanent residence.

"The name of no one but the successful candidate will be made public.

"M. C. JOHNSON, Ch'm. B. T. T. U.

"Lexington, Sept. 20, 1845."

"*The Vacant Chair of Obstetrics.*—The above refers to a *permanent* appointment. The duties of the chair, for the coming session, will be performed by the Professor of Materia Medica and Therapeutics, Dr. Mitchell."

